# Deep sequencing of the microRNA expression in fall dormant and non-dormant alfalfa

**DOI:** 10.1016/j.gdata.2014.09.007

**Published:** 2014-09-28

**Authors:** Wenna Fan, Pengfei Shi, Chengzhang Wang

**Affiliations:** College of Animal Science and Veterinary Medicine, Henan Agricultural University, Zhengzhou, Henan 450002, China

**Keywords:** Deep sequencing, MicroRNA, Fall dormancy, Alfalfa

## Abstract

MicroRNAs (miRNAs) play a critical role in post-transcriptional gene regulation that down-regulates target genes by mRNA degradation or translational repression. Evidence is increasing for their crucial roles during plant development. Identification of miRNAs at the global genome-level by high-throughput sequencing is essential to functionally characterize miRNAs in plants. Alfalfa (*Medicago sativa* L.) is one of the most widely cultivated perennial forage legumes worldwide. Fall dormancy is an adaptive character related to the biomass production and winter survival in alfalfa. However, little is known about miRNA-mediated developmental regulation of fall dormancy in alfalfa. Here, we provide detailed experimental methods and analysis pipeline in our study to identify miRNAs that were responsive to fall dormancy (Fan W et al., Genome-wide identification of different dormant Medicago sativa L. microRNAs in response to fall dormancy, submitted for publication) for reproducible research. The data generated in our work provide meaningful information for understanding the roles of miRNAs in response to seasonal change and growth regulation in alfalfa.

**Table:** 

Specifications	
Organism/cell line/tissue	*Medicago sativa* L. standard varieties Maverick and CUF101
Sex	N/A
Sequencer or array type	Illumina Hiseq 2000
Data format	Raw data: FASTQ files
Experimental factors	Fall dormant vs. nondormant
Experimental features	Small RNA sequencing for miRNA expression analysis in fall dormant and fall non-dormant alfalfa leaves
Consent	N/A
Sample source location	The Experimental Station of Henan Agricultural University, Zhengzhou, China (34°19 × N, 113°35 × E)

## Direct link to deposited data

Deposited data can be found here: http://www.ncbi.nlm.nih.gov/Traces/sra/?study=SRP040470

## Experimental design, materials and methods

### Experimental design

Though the genome sequence of *Medicago truncatula* has been completed, which is an annual legume species with a small diploid genome and easy transformation and is a model plant to study functional genomics of alfalfa, the physiological, biochemical and molecular mechanisms causing fall dormancy (FD) are not clear. Gene regulation expression can be achieved at transcriptional, post-transcriptional and translational levels. In plants and animals, gene silencing occurs when endonuclease complexes are guided by small RNAs to target RNAs. Then exploring the miRNA expression of dormant and non-dormant alfalfa may be important for understanding the mechanism of FD in alfalfa.

### Plant materials and total RNA isolation

Maverick and CUF101 (Alfalfa standard varieties) were planted at the Experimental Station of Henan Agricultural University, Zhengzhou, China. Samples of two different alfalfa varieties in May and September were named DM (Maverick, dormancy—May), NM (CUF101, non-dormancy—May), DS (Maverick, dormancy—September), NS (CUF101, non-dormancy—September), respectively (Table 1).

Alfalfa leaves were collected from Maverick and CUF101, both on May 19 and September 23, 2011, which was 14 days after cutting. Leaves were frozen in liquid nitrogen and then stored at − 80 °C until total RNA isolation. Total RNA was isolated using the TRIzol reagent (Invitrogen, Carlsbad, CA, USA) according to the manufacturer's instructions. RNA degradation and contamination was monitored on 1% agarose gels. The RNA samples were measured with an ND 1000 spectrophotometer (Nanodrop) for contamination with either protein (A260 nm/A280 nm ratio) or reagent (A260 nm/A230 nm ratio).

### Generation of sequencing data

Sequencing libraries were generated using NEBNext® Multiplex Small RNA Library Prep Set for Illumina® (NEB, New England Biolab, and Ipswich, MA, USA.). Briefly, NEB 3′ SR adaptor was directly and specifically ligated to 3′ end of miRNA. Then the 3′ ligation reaction hybridized the SR RT primer. The SR RT primer was hybridized to the excess 3′ SR adaptor (the adaptor remaining free after the 3′ ligation reaction) and transformed it from a single-stranded DNA adaptor into a double-stranded DNA (dsDNA) molecule. The dsDNAs were not substrate for ligation mediated by T4 RNA ligase and therefore did not ligate to the 5′ SR adaptor during the subsequent ligation step. Then a 5′ end adapter was ligated to the 5′ ends of miRNAs. First-strand cDNA was synthesized using M-MuLV Reverse Transcriptase (RNase H–). The cDNA was then PCR amplified using SR primer for Illumina and index primer which contain one of 12 index sequences. The products that were purified (6% TBE PAGE gel, 100 V, 60 min), DNA fragments from 140 to 160 bp (the length of miRNA inserts plus the 3′ and 5′ adaptors), were recovered in a 10 μL elution buffer and quantified using an Agilent high-sensitivity DNA assay on an Agilent Bioanalyzer 2100 system. The clustering of the index-coded samples was performed on a cBot Cluster Generation System using TruSeq SE Cluster Kit v3-cBot-HS (Illumina) according to the manufacturer's instructions. After cluster generation, the library preparations were sequenced on an Illumina Hiseq 2000 platform and samples were run side by side. The raw data have been submitted to NCBI (accession number SRP040470).

### MiRNAs expression analyses

Clean data (clean reads) were obtained by removing low-quality reads and reads containing ploy-N, 5′ primer contaminants, containing poly-A, T, G, or C sections, those without 3′ primers or insert tags from raw data. The Q20, Q30, and GC-content of the raw data were calculated. Then, ranges of length from clean reads were chosen for downstream analysis. The small RNA tags were mapped to a reference sequence using Bowtie [1] without mismatch to analyze their expression and distribution on the reference. Mapped small RNA tags were used to identify the known miRNA in alfalfa. MiRBase 20.0 was used as a reference to modify software mirdeep2 [2] and sRNA-tools-cli was used to obtain the potential miRNA and draw the secondary structures.

The characteristics of hairpin structure of miRNA precursor can be used to predict novel miRNA. The software packages miREvo and mirdeep2 were integrated to predict novel miRNA by evaluating the secondary structure [2], [3]. The Dicer cleavage site and the minimum free energy of the small RNA tags were unannotated in the initial steps. In the current analysis pipeline, known miRNA was used with miFam.dat (http://www.mirbase.org/ftp.shtml) to look for families; novel miRNA precursors were submitted to Rfam (http://rfam.sanger.ac.uk/search/) to look for Rfam families.

**Fig. 1:**
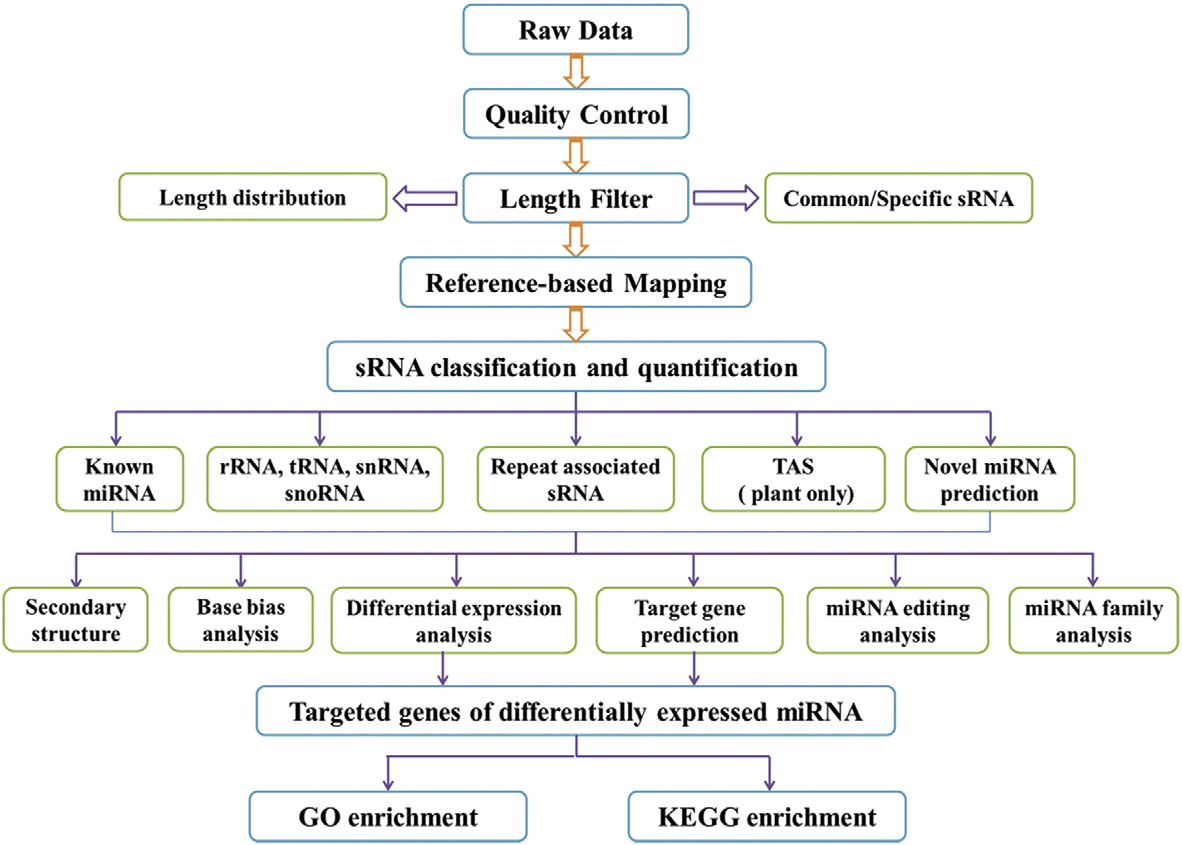
Small RNA analysis process of alfalfa.

The expression levels of miRNA were estimated by transcript per million (TPM) through the following criteria Normalization formula: Normalized expression = mapped read count / Total reads × 10^6^ [4]. Differential expression analyses of four conditions/groups were performed using the DESeq R package. The P-values was adjusted using the Benjamini & Hochberg method. Corrected P-value of 0.05 was set as the threshold for significantly differential expression. P-values were adjusted using Q-value. Q-value < 0.01 and |log_2_(fold change)| > 1 were set as the threshold for significantly differential expression by default [5]. Predicting the target gene of miRNA was performed by psRNATarget (http://bioinfo3.noble.org/psRNATarget/) and psRobot_v1.2 [6], [7]. Gene ontology (GO) enrichment analysis was used on the target gene candidates of differentially expressed miRNAs [8]. KEGG pathways were used to assess the statistical enrichment of the target gene candidates via KOBAS software [9], [10], [11]. The small RNA analysis process of alfalfa was depicted in Fig. 1.

## Discussion

These miRNA-mediated networks could play crucial roles during the dormancy of alfalfa, and our miRNA data provides valuable information regarding further functional analysis of miRNAs involved in fall dormancy of alfalfa. This miRNA resource, combined with previous studies on fall dormancy research in alfalfa, will help us get a closer look at fall dormancy regulation network in alfalfa.

## Conflict of interest

The authors declare no conflict of interest.

## Figures and Tables

**Table 1:** Samples used for sequencing.

Variety	May	September
Maverick (FDC1)	DM	DS
CUF101 (FDC9)	NM	NS

Abbreviations: FDC, fall dormancy class, ranging from 1 to 11, to determine whether a variety is fall dormant type or not.

In this study, FDC1 represents fall dormant type and FDC9 represents fall non-dormant type.
